# Challenging Diagnostic Workup of a 22-year-old Patient With Primary Pigmented Nodular Adrenocortical Disease

**DOI:** 10.1210/jcemcr/luae174

**Published:** 2024-10-01

**Authors:** Jakob Wernig, Stefan Pilz, Christian Trummer, Verena Theiler-Schwetz, Lisa Maria Schmitt, Oleksiy Tsybrovskyy

**Affiliations:** Division of Endocrinology and Diabetology, Department of Internal Medicine, Medical University of Graz, 8010 Graz, Austria; Division of Endocrinology and Diabetology, Department of Internal Medicine, Medical University of Graz, 8010 Graz, Austria; Division of Endocrinology and Diabetology, Department of Internal Medicine, Medical University of Graz, 8010 Graz, Austria; Division of Endocrinology and Diabetology, Department of Internal Medicine, Medical University of Graz, 8010 Graz, Austria; Division of Endocrinology and Diabetology, Department of Internal Medicine, Medical University of Graz, 8010 Graz, Austria; Diagnostic and Research Institute of Pathology, Medical University of Graz, 8010 Graz, Austria

**Keywords:** primary pigmented nodular adrenocortical disease, PPNAD, hypercortisolism, adrenal vein sampling, carney complex

## Abstract

Primary pigmented nodular adrenocortical disease (PPNAD) is a rare cause of ACTH-independent Cushing syndrome (CS), presenting diagnostic challenges due to its rarity and its difficult clinical differentiation from other causes of CS. Here, we report the case of a 22-year-old female who developed classical symptoms of hypercortisolism including progressive weight gain, moon facies, and various skin manifestations. Despite biochemical screening confirming ACTH-independent CS, imaging modalities including computed tomography and magnetic resonance imaging showed normal adrenal gland morphology, complicating the localization of cortisol hypersecretion. Subsequent nuclear imaging methods were not indicative of ectopic cortisol production until adrenal vein sampling (AVS) conclusively identified the adrenal glands as the only possible source of cortisol hypersecretion. Eventually, bilateral adrenalectomy led to a significant improvement in symptoms. Pathological examination confirmed the diagnosis of PPNAD, and genetic testing revealed a mutation in the *PRKAR1A* gene associated with the Carney complex. This case highlights the importance of considering rare etiologies in hypercortisolism diagnosis and describes their challenging diagnostic workup and the utility of AVS in localizing cortisol hypersecretion in PPNAD patients.

## Introduction

Endogenous Cushing syndrome (CS) is mostly caused by excessive ACTH production from pituitary tumors. However, approximately 30% of CS cases are classified as ACTH-independent [1]. Primary pigmented nodular adrenocortical disease (PPNAD) is one of the rarest causes of ACTH-independent CS [2]. While there is consensus that ACTH-independent CS should be treated by surgical removal of 1 or both adrenal glands, there is still a lack of clarity on how to effectively identify PPNAD in patients that present with symptoms of hypercortisolism [1].

The definitive diagnosis of this disease can only be ultimately achieved via pathological examination of the adrenal glands. However, choosing the right clinical diagnostic pathway can lead to a faster diagnosis and therefore more timely and effective treatment.

We report a 22-year-old female patient who initially presented with typical symptoms of hypercortisolism and was eventually diagnosed and treated for PPNAD.

## Case Presentation

A 22-year-old female presented to an outpatient clinic for the first time with the chief complaints of progressive weight gain since the age of 15, oligomenorrhea, and severe acne. Primary assessments included standard laboratory tests for CS (midnight serum cortisol, 24-hour urinary free cortisol, ACTH), abdominal computed tomography (CT) and cranial magnetic resonance imaging (MRI). Laboratory test results led to the diagnosis of ACTH-independent CS, and the patient was admitted to our endocrinology outpatient clinic.

## Diagnostic Assessment

**Figure 1.**
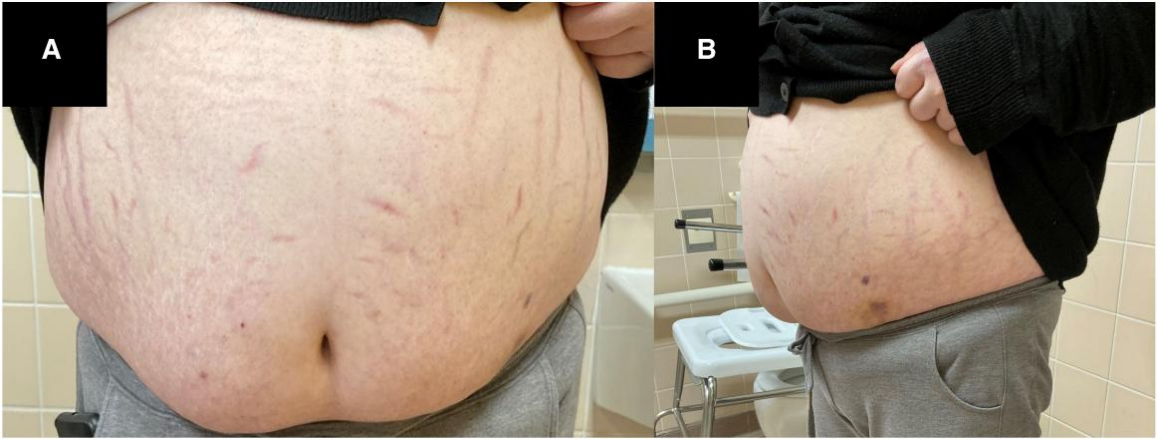
(A) Our patient at first examination presenting herself with truncal obesity, abdominal striae, and multiple hematomas. (B) The truncal obesity contrasts with the slim legs of the patient.

**Figure 2.**
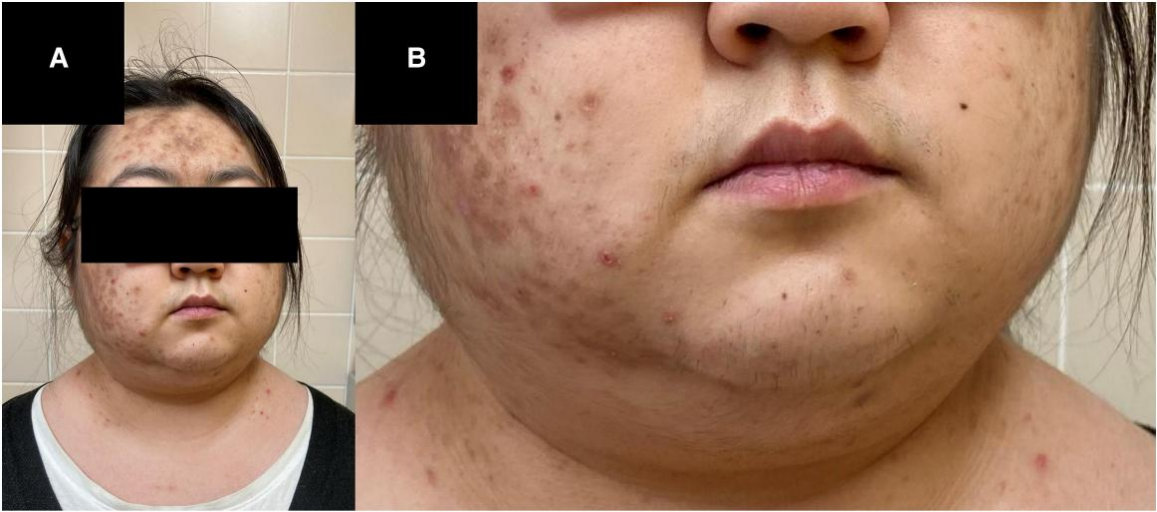
(A) The patient presented herself with moon facies and severe acne. (B) A closer look reveals growth of hair on the upper lip and other parts of the face indicative of hirsutism.

The physical examination revealed thin extremities along with truncal obesity, abdominal striae, and easy skin bruising [Fig. 1]. In addition, moon facies, facial acne, and hirsutism were noticeable [Fig. 2].

We obtained a detailed medical history, which revealed that the patient did not have her menarche until the age of 17. Since then, she has had a normal menstrual cycle until 1 year before consultation, when oligomenorrhea started to occur. Through the medical history, we also identified that the patient had a pulmonary artery embolism 1 year ago. Since then, she has been on rivaroxaban for prophylaxis.

Arterial hypertension had also been diagnosed at the peripheral hospital. At the time of examination, the patient had discontinued the recommended medication and was thus still hypertensive (170/140 mmHg).

Initial biochemical workup is noted in Table 1 and revealed elevated midnight serum cortisol levels of 15.5 μg/dL (428 nmol/L) (normal reference range: 2.7-10.5 μg/dL; 74-291 nmol/L) and a suppressed ACTH concentration of less than 1.6 pg/mL (0.4 pmol/L) (normal reference range: 4.7-48.8 pg/mL; 1.0-10.7 pmol/L). These findings confirmed the presence of ACTH-independent hypercortisolism. Dehydroepiandrosterone sulfate was measured in the outpatient clinic at initial presentation of the patient at 2.07 µg/mL (5.62 µmol/L) (normal reference range: 0.46-2.75 µg/mL; 1.25-7.46 µmol/L). Furthermore, a 1 mg overnight dexamethasone-suppression test was performed and showed morning serum cortisol levels at 28.9 μg/dL (797.2 nmol/L) (normal reference range: < 1.8 μg/dL; < 49.7nmol/L). The 24-hour UFC levels were also elevated at 402.7 µg/24 hours (1111.5 nmol/24 hours) (normal reference range: 20.9-292.3 µg/24 hours; 57.7-806.7 nmol/24 hours). Consistent with the patient's symptoms, laboratory results also showed elevated total testosterone at 0.97 ng/mL (3.36 nmol/L) (normal reference range: 0.14-0.77 ng/mL; 0.49-2.67 nmol/L) and free testosterone at 7.88 pg/mL (27.3 pmol/L) (normal reference range: 0.45-2.19 pg/mL; 1.6-7.6 pmol/L) combined with low SHBG at 1.0 μg/mL (10.6 nmol/L) (normal reference range: 1.8-11.1 μg/mL; 19.0-117.0 nmol/L), biochemically confirming hyperandrogenism. In addition, laboratory findings revealed elevated cholesterol levels of 254 mg/dL (6.6 mmol/L) (normal reference range: < 200 mg/dL; 5.2 mmol/L) and triglycerides at 207 mg/dL (2.3 mmol/L) (normal reference range: < 150 mg/dL; < 1.7 mmol/L), indicative of combined hyperlipidemia.

**Table 1.** Laboratory findings on initial presentation of the patient to confirm Cushing syndrome including other abnormal findings

Serum labs (time point)	Initial presentation	Reference range
Serum cortisol (23:00)	**15.5 μg/dL** **(428 nmol/L)**	2.7-10.5 μg/dL (74-291 nmol/L)
ACTH (23:00)	**<1.6 pg/mL** **(0.4 pmol/L)**	4.7-48.8 pg/mL (1.0-10.7 pmol/L)
Serum cortisol after 1 mg dexamethasone-supression test (08:00)	**28.9 μg/dL** **(797.2 nmol/L)**	<1.8 μg/dL (<49.7 nmol/L)
DHEA-S	2.07 µg/mL (5.62 µmol/L)	0.46-2.75 µg/mL (1.25-7.46 µmol/L)
24-h UFC	**402.7 µg/24h** **(1111.5 nmol/24 hours)**	20.9-292.3 µg/24h (57.7-806.7 nmol/24 hours)
Total testosterone	**0.97 ng/mL** **(3.36 nmol/L)**	0.14-0.77 ng/mL (0.49-2.67 nmol/L)
Free testosterone	**7.88 pg/mL** **(27.3 pmol/L)**	0.45-2.19 pg/mL (1.6-7.6 pmol/L)
SHBG	**1.0 μg/mL** **(10.6 nmol/L)**	1.8-11.1 μg/mL (19.0-117.0 nmol/L)
Total cholesterol	**254 mg/dL** **(6.6 mmol/L)**	<200 mg/dL (<5.2 mmol/L)
Triglycerides	**207 mg/dL** **(2.3 mmol/L)**	<150 mg/dL (< 1.7 mmol/L)

Abnormal values are shown in bold font. Values in parenthesis are International System of Units.

Abbrevations: DHEA-S, dehydroepiandrosterone sulfate; 24-h UFC, 24-hour-urinary free cortisol.

To check for possible osteoporosis, we performed a bone density test (dual-energy X-ray absorptiometry scan). The Z-scores were −3.5 at the lumbar spine (L1-L4), −3.2 at the femoral neck, and −2.7 at the total hip region.

In a subsequently performed abdominal CT, both adrenal glands appeared normal in size and structure. They were also unremarkable in an abdominal MRI, thus raising the question whether there was an adrenal or extra-adrenal source of cortisol hypersecretion.

Therefore, we performed nuclear imaging methods, but both ^18^F-DOPA positron emission tomography-computed tomography (PET-CT) and ^68^Gallium-DOTANOC PET-CT showed no pathologically elevated tracer uptake in the adrenal glands or other parts of the body.

As the aforementioned diagnostic procedures did not reveal the origin of hypercortisolism, we were left with 2 differential diagnoses: bilateral micronodular hyperplasia or the existence of an ectopic cortisol-producing tumor of unknown origin. Based on these considerations, we decided to perform adrenal vein sampling (AVS) to localize the source of cortisol production. AVS of both adrenal veins, renal veins, and ovarian veins was performed. Results did not indicate any extra-adrenal secretion of cortisol and therefore confirmed the adrenal glands as the only possible origin of cortisol overproduction. As shown in Table 2, it was apparent that both glands contributed to the hypersecretion of cortisol. In conclusion, the most likely differential diagnosis left was PPNAD, which is treated by bilateral adrenalectomy.

**Table 2.** Adrenal vein sampling results

Site	Cortisol	Reference range
Peripheral blood sample	**26.4 μg/dL** **(728.6 nmol/L)**	5.3-22.5 μg/dL (146.3-621 nmol/L)
Left ovarian vein	**24.9 μg/dL** **(687.2 nmol/L)**	5.3-22.5 μg/dL (146.3-621 nmol/L)
Right ovarian vein	20.9 μg/dL (577.4 nmol/L)	5.3-22.5 μg/dL (146.3-621 nmol/L)
Left renal vein	21.9 μg/dL (604.2 nmol/L)	5.3-22.5 μg/dL (146.3-621 nmol/L)
Right renal vein	18.5 μg/dL (511.4 nmol/L)	5.3-22.5 μg/dL (146.3-621 nmol/L)
Left adrenal vein	**240.4 μg/dL** **(6631.0 nmol/L)**	5.3-22.5 μg/dL (146.2-620.7 nmol/L)
Right adrenal vein	**146.2 μg/dL** **(4033.6 nmol/L)**	5.3-22.5 μg/dL (146.2-620.7 nmol/L)

Abnormal values are shown in bold font. Values in parenthesis are International System of Units.

## Treatment

Our endocrine surgery department successfully performed bilateral adrenalectomy. After that intervention, a lifelong adrenal hormone replacement therapy that consists of glucocorticoids and mineralocorticoids is mandatory [3, 4]. We started the hormone replacement therapy on the first day after surgery using 30 mg of hydrocortisone and 0.05 mg of fludrocortisone.

Based on the dual-energy X-ray absorptiometry scan results and considering the absence of previous fractures and a relatively low fracture risk at this young age, we opted not to prescribe osteoporosis medication, but we did recommend adequate calcium and vitamin D supplementation.

## Outcome and Follow-up

**Figure 3.**
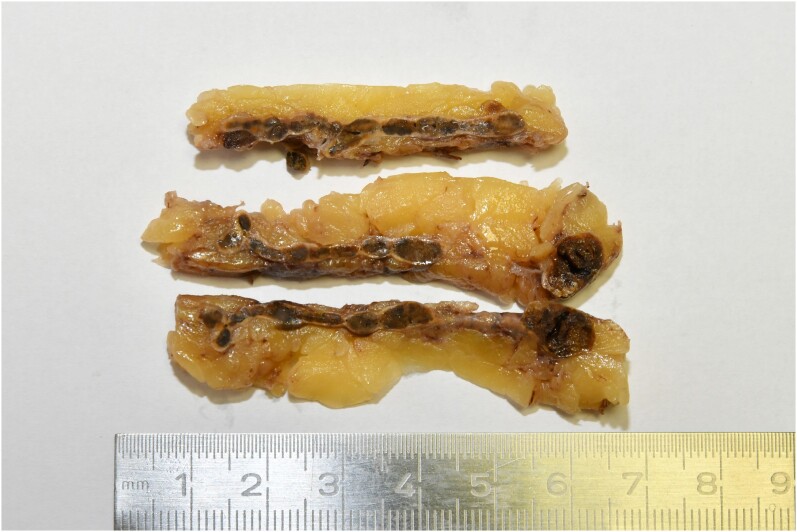
Adrenalectomy specimen (formalin fixed) showing multiple micro-nodules (< 1 cm diameter) in the adrenal cortex on cut surface. This is the typical macroscopic appearance of primary pigmented nodular adrenocortical disease.

The final diagnosis of PPNAD was eventually confirmed by histopathology. The typical brown hyperpigmentation was evident on gross visual inspection [Fig. 3].

**Figure 4.**
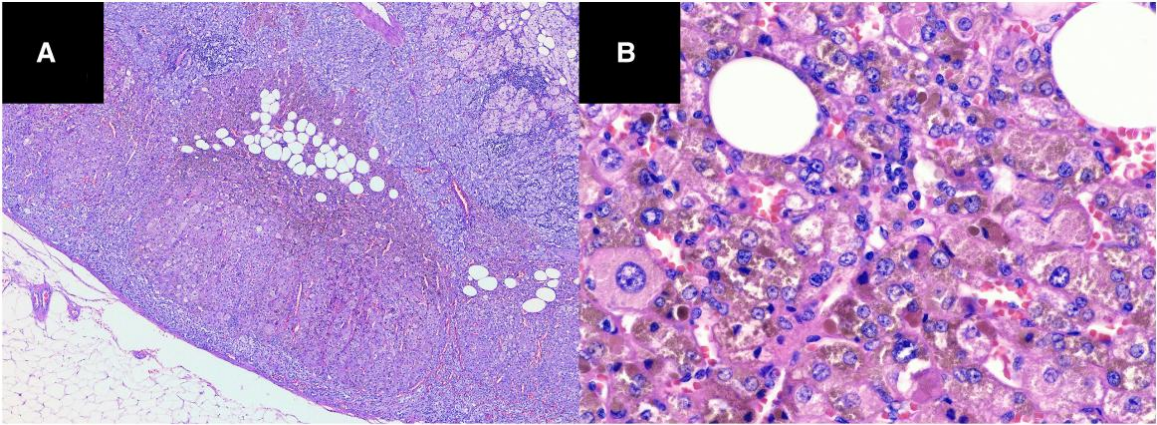
(A) Histology of the adrenalectomy specimen (hematoxylin and eosin stain). On low power, one can recognize multiple micro-nodules in the adrenal cortex; most of them are associated with the islands of fat tissue. (B) On high power, the cells within the nodules show pronounced anisokaryosis, variable enlargement of the cytoplasm, and marked accumulation of pigment.

Within the adrenal cortex, multiple nodules could be found with the largest extend of 1 cm. Histology also revealed a marked accumulation of brown pigment [Fig. 4].

Shortly after, we decided to perform genetic analysis. Via next-generation sequencing, a mutation of the *PRKAR1A* gene (heterozygote) was discovered. In our patient, the exact variant of the mutation is *c.3G > A, p.(? )*, which was deemed likely pathogenic for Carney complex (CNC), although it has not yet been described in international databases or the literature. The mutation *c.3G > A* disrupts the start codon methionine, preventing proper translation of the protein [5].

In the clinical examination, no skin manifestations of CNC were observed. However, several other findings potentially associated with CNC could be identified. Initially, it was found that the patient had suffered a pulmonary artery embolism 1 year before presentation. Although rare, cases have been documented where a cardiac myxoma precipitates the pulmonary artery embolism [6, 7]. Echocardiography and CT scan of the chest at the time of diagnosis did not detect a myxoma. Furthermore, ultrasound imaging revealed the presence of an ovarian cyst, and thoracic MRI detected a contrast-enhancing nodular lesion in the right breast. These findings are both plausible clinical manifestations in CNC patients [8].

At last follow-up, 5 months after surgery, the patient presented at our outpatient clinic with clear improvement in symptoms. Her skin condition had improved significantly. In addition, she had lost an impressive 20 kg since surgery. According to the patient, her menstrual cycle had also returned to normal. The patient learned to correctly perform the glucocorticoid replacement therapy on her own and was given an emergency hydrocortisone kit. Ongoing regular check-ups in our outpatient clinic are performed to monitor the replacement therapy and CNC-associated pathologies such as myxomas or skin lesions.

## Discussion

First, the main challenge for our diagnostic workup of ACTH-independent hypercortisolism was the unremarkable morphology of both adrenal glands in CT and MRI. In a study by Vezzosi et al, the adrenal CT scan results of 17 PPNAD patients exhibited a spectrum of findings including normal, micronodular, or macronodular imaging. Notably, only 3 of 17 patients in the Vezzosi study and 9 of 33 in the Powell study had unremarkable adrenal glands [9, 10]. Therefore, any undefining results of CT in patients with ACTH-independent CS should suggest the differential of PPNAD, but ectopic sources of cortisol hypersecretion may also be present.

Nuclear imaging can be used to detect ectopic cortisol production. A 10-year-long retrospective study by Zisser et al stated the best results with Gallium-DOTANOC PET-CT and ^18^F-DOPA, both of which were performed in our patient. However, it must be noted that the sensitivity was also dependent on the etiology of CS [11]. Another published case describes a 57-year-old patient where an ectopic adrenocortical adenoma was detected in the right pulmonary hilus via ^68^Gallium PET-CT [12].

In our case, AVS was the decisive diagnostic test. AVS is commonly used in patients with primary aldosteronism [13]. Cases in which AVS is used in patients with hypercortisolism are rare. At the current time, there is no consensus on the definition of lateralization in this context, which is why we interpreted AVS in analogy to primary aldosteronism. Additionally, cutoff values are not yet standardized [14]. However, by measuring cortisol levels in the adrenal vein and a peripheral vein, AVS can identify unilateral excess cortisol secretion, helping in the localization of autonomous cortisol-secreting adenomas or hyperplasia. This technique allows for a more targeted and precise approach in the management of patients with bilateral adrenal masses or ACTH-independent CS [15].

A recent study has reported promising results in standardizing cortisol ratios using the inferior vena cava as a reference value. This method has shown positive outcomes in distinguishing between unilateral and bilateral cortisol hypersecretion [16]. Furthermore, another study demonstrated that AVS can determine unilateral excess cortisol production through the difference in cortisol levels between the left and right adrenal veins, achieving a success rate of 85% in the procedures conducted. Interestingly, study results also revealed that nearly half of the patients diagnosed with unilateral tumors on CT show bilateral overproduction of cortisol [17].

This indicates the potential role of AVS in determining whether both adrenal glands need to be removed or if unilateral adrenalectomy is sufficient.

In a similar case of a 40-year-old patient, ovarian and adrenal vein sampling discovered an ovarian origin of cortisol secretion, leading to a laparoscopic salpingo-oophorectomy of the right-side ovary [18]. Another published case featured a 21-year-old female presenting with similar symptoms to our patient. CT imaging showed tiny nodules on her left adrenal gland, which were then verified by elevated plasma cortisol in the left adrenal vein using AVS [19]. In our patient, cortisol levels in both adrenal veins were distinctly elevated (Table 2). Considering the undefining imaging results, we concluded that both adrenal glands would be affected by the disease; this was later confirmed by the pathological examination.

Second, postoperative genetic examination showed the presence of CNC in our patient. CNC is a rare genetic syndrome that is mainly caused by mutations of the *PRKAR1A* gene [20]. Sun et al provide a comprehensive systematic review summarizing the pathogenic variants and clinical features identified in 210 PPNAD patients [21]. PPNAD and CNC are strongly related to each other as shown by Bertherat et al. In only 12% of PPNAD patients, CNC was not detected [22]. As CNC has various manifestations, including endocrine organ diseases, skin lesions, and cardiac myxomas, it is imperative to take CNC into consideration when planning the follow-up [23].

## Learning Points

- In patients with confirmed ACTH-independent CS and normal adrenal gland morphology in CT or MRI, the possibility of PPNAD should be suggested.
- AVS can be a valid option to detect the origin of cortisol hypersecretion.
- AVS should be performed if other imaging methods fail to localize the origin of cortisol hypersecretion.
- PPNAD patients should always be screened for the existence of CNC via genetic testing.

## Contributors

All authors made individual contributions to the authorship of this work. J.W. was involved in manuscript drafting. S.P. and C.T. were involved in the diagnosis and management of the patient. S.P., C.T., V.T., and L.S. were involved in manuscript editing and in the critical review of the manuscript. O.T. was involved in the histopathology section and preparation of histology images. All authors reviewed and approved the final draft.

## Data Availability

Original data generated and analyzed during this study are included in this published article.
